# Intragastric Rupture of a Splenic Artery Aneurysm Associated with a Pancreatic Cancer

**DOI:** 10.5334/jbr-btr.1008

**Published:** 2016-02-10

**Authors:** Aman Toukouki, Nicolas Verbeeck, Jos Weber, Vincent Lens

**Affiliations:** 1 Department of Radiology, Centre Hospitalier de Luxembourg, 4, Rue Barblé, L-1210 Luxembourg, Grand Duchy of Luxembourg; 2 Department of Gastroenterology, Centre Hospitalier de Luxembourg, 4, Rue Barblé, L-1210 Luxembourg, Grand Duchy of Luxembourg

**Keywords:** Digestive hemorrhage, Splenic artery aneurysm, Pancreatic neoplasm, Angiography, Computed tomography

## Abstract

Acute upper digestive tract hemorrhage most often arises from gastric and esophageal vessels located in the mucosa or the submucosa. Rupture in the upper gastrointestinal tract is a classical but uncommon complication of arterial (mainly the abdominal aorta) aneurysms. Splenic artery aneurysm usually ruptures in the peritoneum, unless it is associated with a disease eroding the gastrointestinal wall. We present and describe the management of the rare occurrence of an intragastric rupture of a splenic aneurysm associated with a pancreatic cancer.

## Publisher’s Note

This article was originally published without the text content, which was added shortly after. The publisher apologizes for this omission.

## Introduction

Acute upper digestive tract hemorrhage (UDTH) is a life-threatening condition with multiple causes, mostly arising from the rupture of vessels in the esophageal or gastric submucosa or mucosa. Patients with UDTH are amenable to endoscopic diagnosis. Then, depending on the cause and the patient’s status, the treatment may include endoscopic surgery or endovascular procedures. When endoscopy fails to contribute to the diagnosis, intravenous contrast-enhanced computed tomography (CT) may help in identifying unsuspected causative lesions and hence facilitate surgical or endovascular treatment. We present an uncommon case of UDTH, where bleeding arose from the intragastric rupture of a splenic artery aneurysm (SAA) after erosion by a pancreatic cancer.

## Case Report

**Figure 1:**
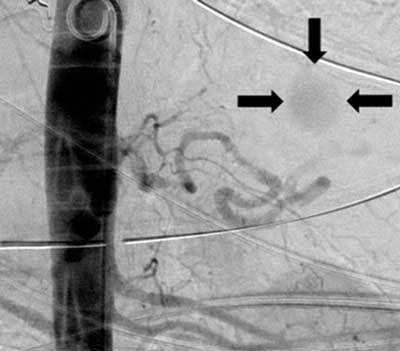
Abdominal aortic angiogram: the 2.5 cm large aneurysm of the distal splenic artery (arrows).

A 61-year-old male was admitted to our hospital for unconsciousness followed by melena. On admission, he remained hemodynamically stable with a hemoglobin level at 12 g/dl (normal values: 14–18 g/dl). The patient underwent an upper endoscopy which revealed a stomach full of fresh blood. Neither esophageal varices nor ulcer were found. The patient rapidly progressed to hemodynamic instability, requiring intubation and polytransfusion. He was then taken to the angiography suite. The global aortic angiogram demonstrated the existence of a saccular aneurysm of 2.5 cm in diameter on the distal third of a splenic artery which also showed irregular contours (Figure 1). This irregularity was not associated with the usual findings of spasticity (Figure 2a). However, it prevented the distal selective catheterization of the artery, using a 3F microcatheter. Given the impossibility of a “sandwich” coiling, we opted for a postostial embolization with microcoils, enabling hemodynamic stabilization (Figure 2b).

**Figure 2a:**
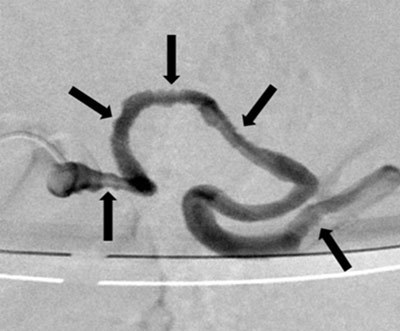
Selective splenic artery opacification: the multiple parietal irregularities (arrows).

**Figure 2b:**
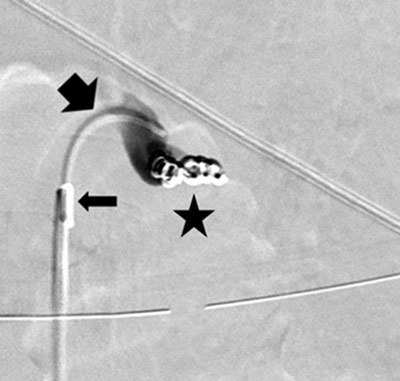
Postostial occlusion of the splenic artery: the 5F support catheter (thin arrow), the 3F microcatheter (thick arrow), and the microcoils (star).

**Figure 3:**
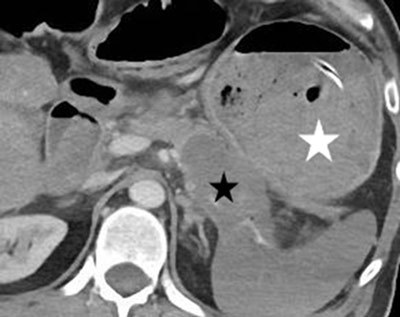
Axial contrast-enhanced CT image: the pancreatic neoplasm (black star) and the almost entirely clotted gastric lumen (white star).

In the wake, a CT scan was performed. A distal pancreatic tumor abutting the posterior gastric wall was demonstrated. We speculate it could be responsible for the splenic artery erosion, with the development and secondary rupture of a false aneurysm in the gastric cavity (Figure 3). The intravenous injection of contrast material did not reveal persisting blood extravasation. A new gastroscopy was performed the next day. As the stomach was no longer filled with blood, a posterior subcardial perforation was discovered, and biopsies were performed on its margins. Histological examination revealed invasive pancreatic ductal adenocarcinoma.

Six days later, the patient presented a slow decrease of his blood hemoglobin (7.4 g/dl). A new contrast-enhanced CT scan revealed a new retrogastric blood extravasation originating from the distal third of the splenic artery (Figure 4a). A superselective catheterization of the gastroepiploic arteries by a 3F microcatheter allowed coil embolization of the distal splenic artery (“sandwich” method in two stages, see infra), hence stopping the bleeding (Figure 4b). After three months of chemotherapy, there was a reduction of the size of the pancreatic adenocarcinoma but also occurrence of liver and bone metastases. The patient died eight months later.

**Figure 4a:**
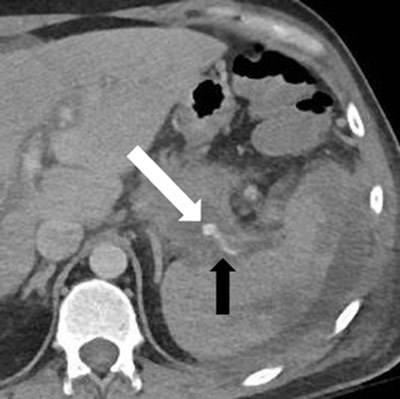
Enhanced CT, a few days later: recurrent hemorrhage (white arrow) arising retrogradely from the distal splenic artery (black arrow).

**Figure 4b:**
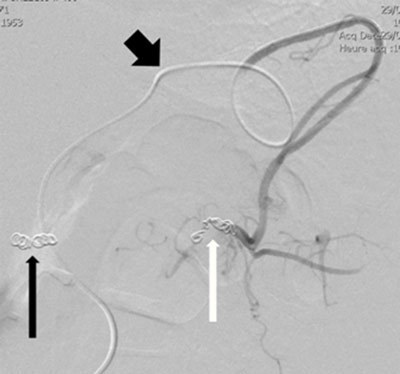
Microcoils embolization of the distal splenic artery (white arrow) through superselective catheterism of the gastroepiploic arteries (thick black arrow); the thin black arrow indicates the previous ostial coiling.

## Discussion

The main causes for severe UDTHs remain gastroduodenal ulcers and esophageal varices in the context of portal hypertension [1]. In rarer cases, massive deglobulinization is due to an aortoduodenal fistula, to the rupture of a hemorrhagic pancreatic pseudocyst in the gastric or duodenal lumen, or to the intragastric rupture of an SAA as reported here [2,3,4,5,6,7,8]. Massive UDTHs, whose mortality rate is about 10 percent, benefit essentially from endoscopic treatment [1]. Whereas the varices responsible for the bleeding are ligated, other hemorrhagic lesions respond to thermal coagulations, adrenaline injection, and hemostatic clip placement [9]. When the bleeding site cannot be located or when the blood losses cannot be controlled by classical means, intravenous enhanced CT scan should be proposed, provided the hemodynamic condition of the patient allows it. This examination will often identify the cause of the bleeding as well as its site and enable an ideal planning of the treatment, whether it be endovascular or surgical [8,10]. Hemodynamically unstable patients must undergo emergency exploratory and therapeutic surgery unless an interventional angiography suite is available; if this is the case, the patient must have access to it [11,12].

Splenic artery aneurysms (SAAs) represent about one fifth of splanchnorenal aneurysms [5]. They are being detected with increasing frequency as a result of the widespread use of cross-sectional imaging techniques [2]. The true aneurysm is the result, among others, of lesions of the elastic tissue and of the smooth muscle cells of the artery wall, lesions which are enhanced by atherosclerosis, arterial hypertension, portal hypertension, fibro-muscular dysplasia, and an increase of estroprogestative hormone levels [2,5,8]. This latter factor explains the frequency of SAAs and their increased fragility in pregnant women [7].

The false aneurysm or pseudoaneurysm occurs in the case of trauma of the artery and has no own artery wall. It results from a direct trauma or, in the case of acute pancreatitis, for example, from a parietal digestion by pancreatic enzymes [2,8]. Most often fortuitously discovered and measuring less than 2 cm, it can grow and cause aspecific symptoms like nausea, vomiting, or pain in the left upper quadrant [2,5,8]. The aneurysm rupture occurs in 2–3 percent of the cases and is burdened by a mortality of 10–25 percent, and even higher in pregnant women [8]. In two thirds of the cases, it will occur in the peritoneal cavity and in the lumen of a hollow organ in the other instances [2].

Our case reports the association of a pancreatic cancer with SAA which ruptured in the stomach. To the best of our knowledge, this is the first such case reported in the medical literature. It is virtually impossible to know whether a true SAA has preceded the neoplasm or if the cancer has induced the formation of a false aneurysm, but the anfractuosity of the splenic artery on angiography makes us rather advocate for the second hypothesis.

SAA rupture in general happens in one or two stages with, in the latter case, a hemorrhage which is first limited to the omental bursa and then extends to the whole peritoneal cavity [7,8]. SAAs derive great benefit from contrast-enhanced CT since they are deeply located and poorly detected by echography. Magnetic resonance should be reserved for nonurgent cases [8]. Whereas ruptures must be treated immediately, there is no recognized consensus about lesions that are discovered incidentally. The medical literature recommends treating women in reproductive age, cases with a fibro-muscular dysplastic origin, and those associated with badly controlled arterial hypertension. Wider than 20 mm aneurysms should also be treated preventively [5].

The ideal endovascular treatment consists in the deployment of a stent graft in the splenic artery at the level of the aneurysm, which may be prevented in some cases by the vessel tortuosity. Another method consists in the placement of a noncovered stent and secondary filling of the aneurysmal sac through the prosthesis. In case of acute emergency, the splenic artery must be embolized in taking care, mainly in the case of proximal SAA and if it is technically feasible, to proceed upstream and downstream the aneurysmal sac (or “sandwich” method) to avoid rebleeding by reperfusion due to collateral blood supply [8]. In the present case, this procedure was completed in two times because of the initial aspect of the splenic artery. The whole procedures allowed patient survival in the range of survival for advanced pancreatic cancer.

## Conclusion

Intragastric rupture of an SAA is a possible but rare etiology of UDTH, especially after gastric erosion by a pancreas cancer, as in the case we have presented. A correct identification of this potentially lethal entity is within the reach of modern CT scanners; it enables a relevant orientation of patients to a well-targeted endovascular or surgical procedure.

## Competing Interests

The authors declare that they have no competing interests.

## References

[1] Paugam-Burtz C, Bonneville C (2008). Hémorragies sévères. Place respective de la chirurgie et de la radiologie interventionnelle. Congrès national d’anesthésie et de réanimation.

[2] Wierzbicki T, Szmeja J, Borejsza-Wysocki M (2012). Massive bleeding from upper gastrointestinal tract as symptom of rupture of splenic artery aneurysm to stomach. Med Sci Monit.

[3] Lesur G (2005). Les causes rares d’hémorragies digestives hautes. Association Française de Formation Médicale Continue en Hépato-Gastro-Entérologie.

[4] Vilcea V, Nemes R, Georgescu I (2001). Exceptional etiologies in upper digestive tract bleeding. Chirurgia.

[5] Cochennec F, Riga CV, Allaire E (2011). Contemporary management of splanchnic and renal artery aneurysms: results of endovascular compared with open surgery from two European vascular centers. Eur J Vasc Endovasc Surg.

[6] Scheiermann P, Meimarakis G, Bamberg F (2012). Ruptured splenic artery aneurysm masquerading as a gastric hemorrhage. J Vasc Surg.

[7] Pavlis T, Seretis C, Gourgiotis S (2012). Spontaneous rupture of splenic artery aneurysm during the first trimester of pregnancy: report of an extremely rare case and review of the literature. Case Rep Obstet Gynecol.

[8] Miao YD, Ye B (2013). Intragastric rupture of splenic artery aneurysms: three case reports and literature review. Pak J Med Sci.

[9] National Institute for Health Clinical Excellence (2012). Acute upper gastrointestinal bleeding: management. www.nice.org.uk.

[10] Zins M (2012). Prise en charge radiologique des hémorragies digestives graves d’origine artérielle. Journées Françaises de Radiologie.

[11] Lesur G, Manière T (2008). Hémorragies digestives graves. Ateliers de la Société Française d’Endoscopie Digestive.

[12] Verbeeck N, Stammet P, Weber J (2009). Bleeding duodenal varices, an unusual presentation of portal hypertension: 3D MSCT of the feeding branches facilitates temporizing treatment by percutaneous transhepatic embolization. JBR-BTR.

